# Cardiac Involvement in a Lymphoma: A Rare Extranodal Site

**DOI:** 10.7759/cureus.79941

**Published:** 2025-03-03

**Authors:** Vino Anand S, Kannan J, Satheesh Kumar

**Affiliations:** 1 Medical Oncology, Madras Medical College, Chennai, IND

**Keywords:** b-cell neoplasm, cardiac involvement, dlbcl, non-hodgkin’s lymphoma, right atrial cardiac mass

## Abstract

Cardiac involvement in lymphoma is rare and usually presents as a late manifestation of the disease. Although clinically asymptomatic and mostly found on autopsy, some patients may present with ventricular outflow obstruction, valve dysfunction, arrhythmias, pericardial effusion, cardiac tamponade, and tumor embolization. Multimodality imaging is important in distinguishing cardiac masses to provide optimal treatment. Here, we describe the case of a 54-year-old woman who presented with dyspnea on exertion and hemodynamic compromise. With the aid of multimodality imaging, the patient was found to have a right atrial cardiac mass. Given her worsening symptoms and hemodynamic instability, the patient underwent excision of the right atrial mass. Ultimately, histopathology revealed diffuse large B-cell lymphoma. The patient was evaluated further and completed systemic therapy without any adverse events and achieved complete metabolic response (Deauville score 1). The patient completed her first-year follow-up, and an annual positron emission tomography-computed tomography showed no evidence of disease.

## Introduction

Cardiac involvement in lymphoma is a late manifestation of the disease. It is associated with 9-20% of lymphoma patients and carries a poor prognosis [1]. The most common primary tumor of the heart is myxoma, followed by papillary fibroelastoma, rhabdomyoma, fibroma, hemangioma, paraganglioma, and lipoma. Sarcoma of the heart represents the second most common neoplasm. Manifestations of benign tumors include mural, intracavitary, and epicardial focal masses. Malignant tumors present with diffuse or infiltrative involvement [2]. Metastases to the heart are more common compared to primary cardiac malignancy. Tumor involvement includes retrograde lymphatic extension, hematogenous spread, and direct contiguous extension [3]. Lymphoma can affect the heart either primarily or secondarily. Patients usually present with extracardiac-related symptoms such as fever, fatigue, weight loss, and drenching night sweats, making it challenging to diagnose cardiac involvement in systemic lymphoma. Here, we report the clinical presentation, treatment modality, and treatment outcomes of cardiac involvement in diffuse large B-cell lymphoma.

## Case presentation

**Figure 1:**
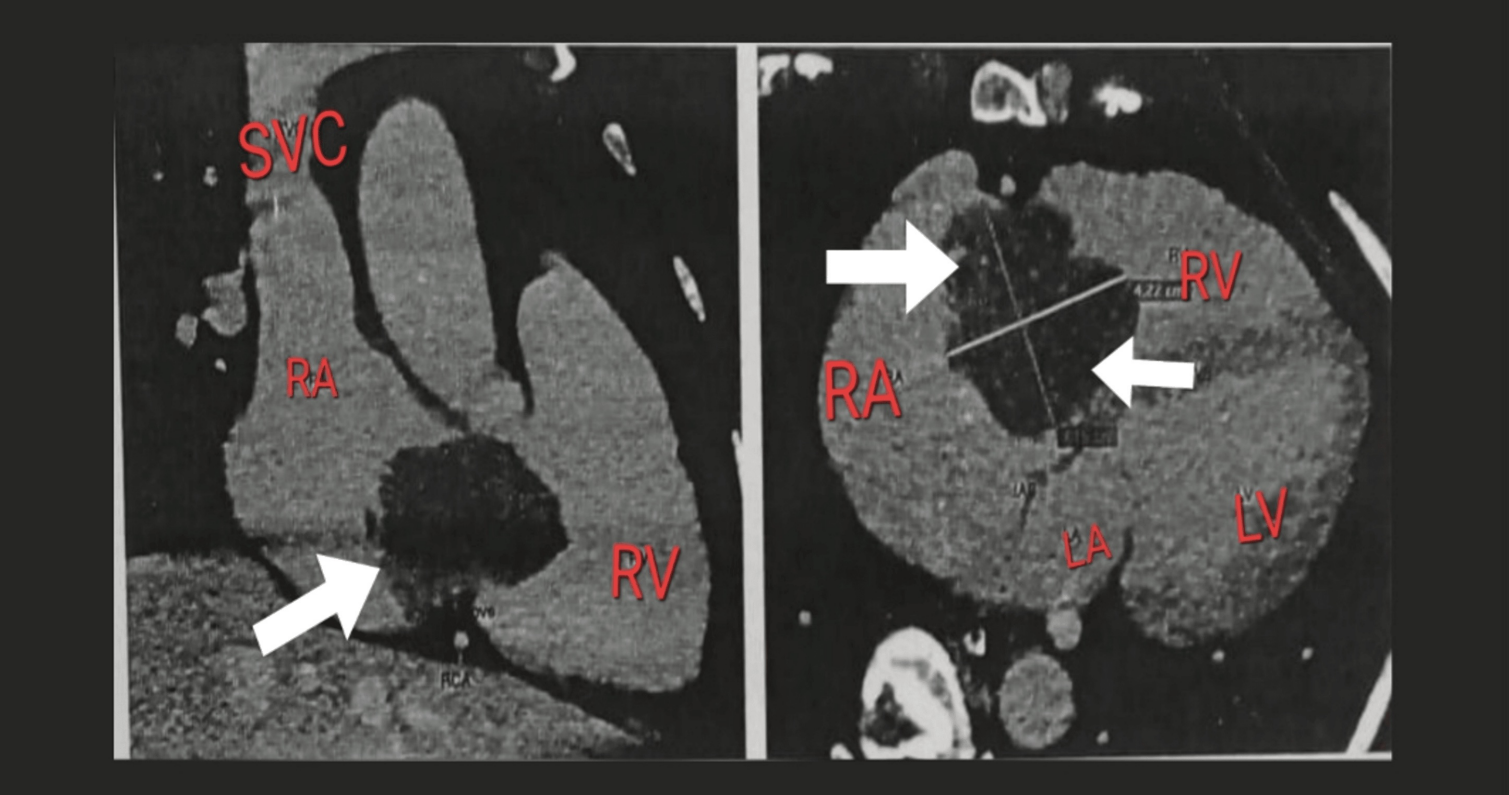
CT cardiac angiogram. The arrow shows the right atrial mass. The figure shows the dilated right atrium measuring 3.3 × 4.2 × 4.5 cm. RA = right atrium; LA = left atrium; RV = right ventricle; LV = left ventricle; SVC = superior vena cava

A 54-year-old female with hypothyroidism presented to the emergency department with dyspnea on exertion, chest discomfort, and tachycardia. On further evaluation, the chest X-ray showed an increased cardiothoracic ratio with increased bronchovascular markings in bilateral lung fields, leading to pulmonary congestion. The echocardiogram showed features suggestive of a mobile mass in the right atrium, dilated right atrium, and mild right ventricular systolic dysfunction. The coronary angiogram suggested a right atrial mass and normal epicardial coronaries. The CT cardiac angiogram showed a dilated right atrium with well-defined lobulated non-enhancing mass lesion in the right atrium measuring 3.3 × 4.2 × 4.5 cm (Figure 1).

**Figure 2:**
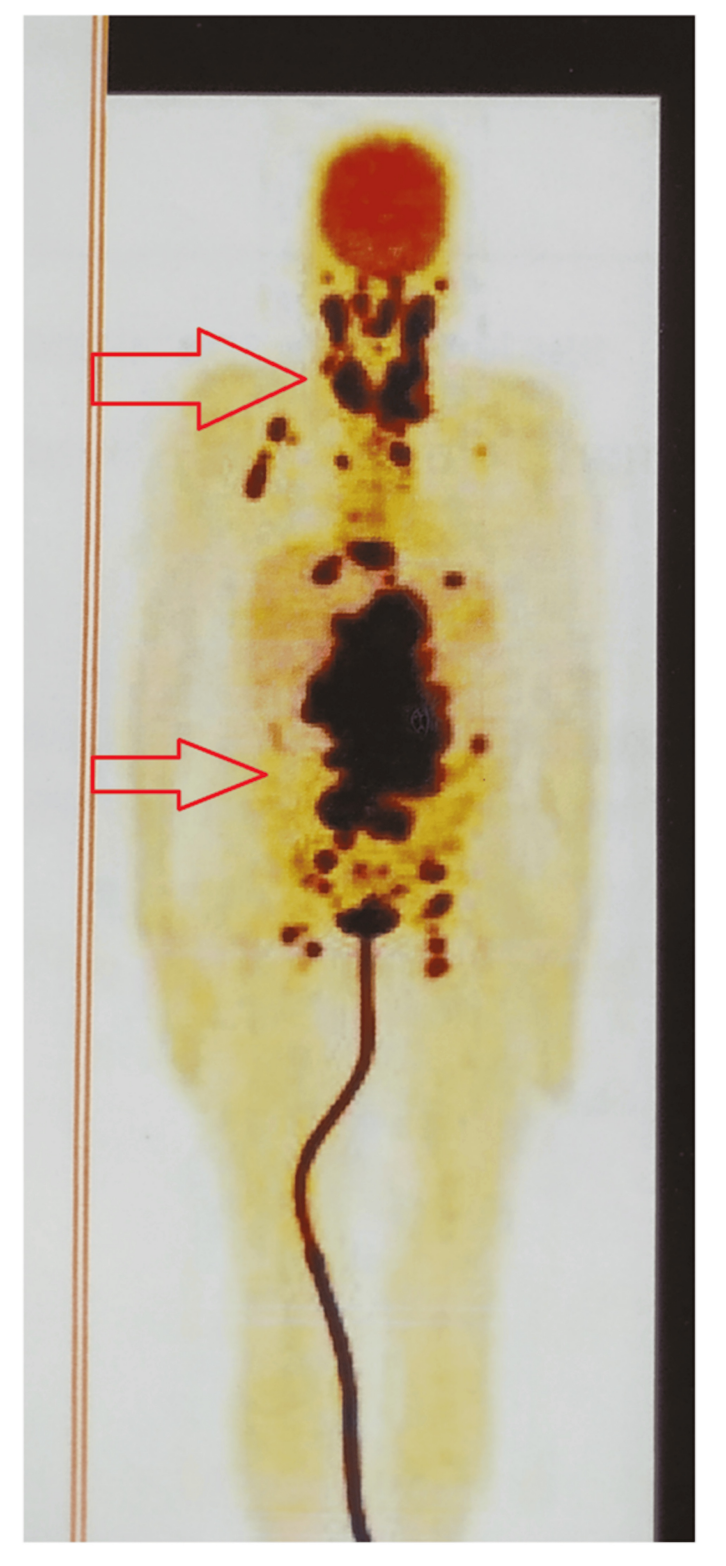
Pre-chemotherapy positron emission tomography-computed tomography scan showing supra and infra-diaphragmatic lymph nodes.

Due to worsening shortness of breath and deteriorating general condition, the patient underwent an excision of the right atrial mass. Postoperatively, the patient’s general condition improved with sinus rhythm on the electrocardiogram and echocardiogram, with an ejection fraction of 64%. Postoperative histopathology suggested non-Hodgkin’s diffuse large B-cell lymphoma, activated B-cell type. Immunohistochemistry markers were positive for leukocyte common antigen, cluster of differentiation 20, B-cell lymphoma 2, and multiple myeloma oncogene 1. Positron emission tomography-computed tomography (PET-CT) had supra and infra-diaphragmatic features with residual lesions in the right atrium (Figure 2).

**Figure 3:**
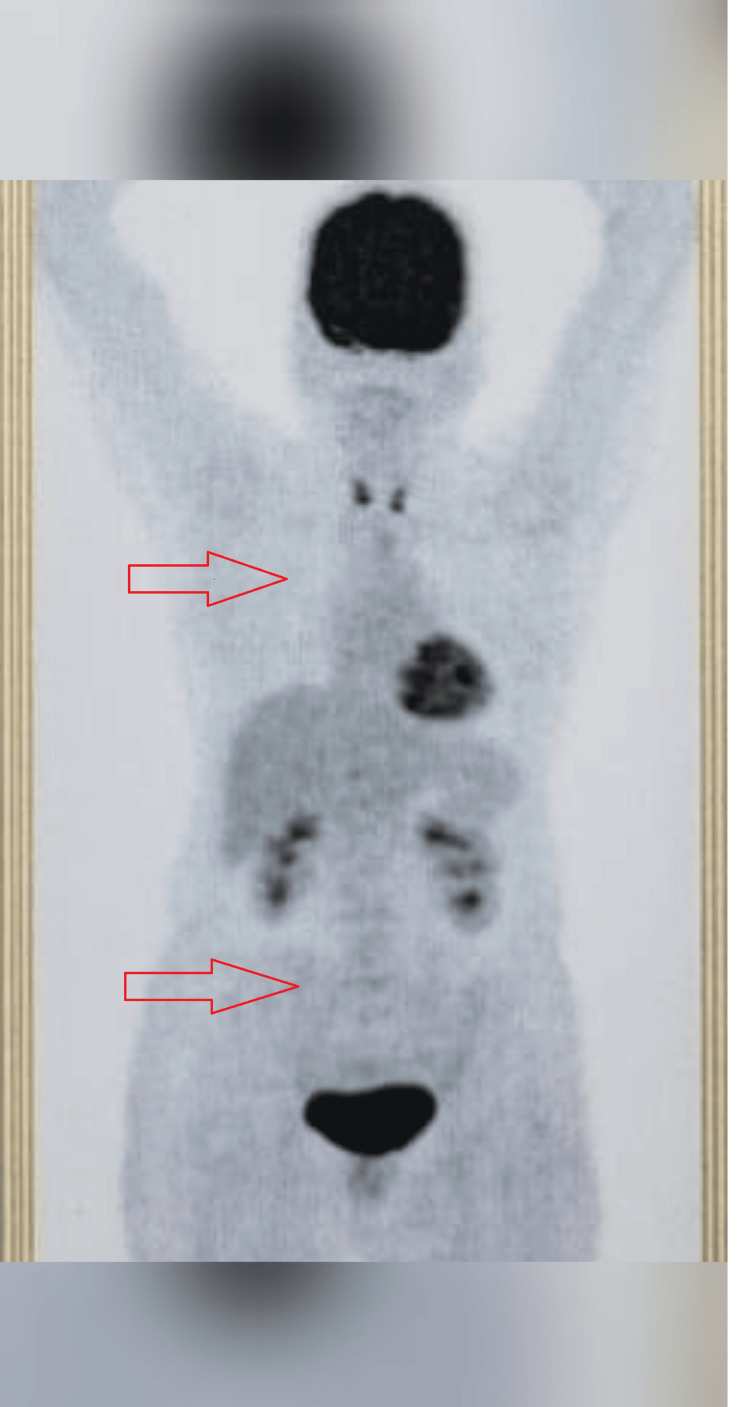
Post-chemotherapy positron emission tomography-computed tomography scan showing complete metabolic response.

Bone marrow aspiration and bone marrow biopsy studies were negative. Blood parameters such as hemogram, renal function, and liver function tests were in the normal range. Serum lactate dehydrogenase was elevated at 580 U/L. The patient was diagnosed with diffuse large B-cell lymphoma stage IV. International Prognostic Index score: Low (intermediate risk score of 1). Central nervous system disease International Prognostic score: Intermediate risk of score 2. The patient started and completed six cycles of R-CHOP chemotherapy. Post-chemotherapy PET-CT showed a complete metabolic response to therapy (Deauville score 1) (Figure 3). Follow-up PET-CT showed no evidence of disease (Figure 3), and an echocardiogram showed an ejection fraction of 65%.

**Figure 4:**
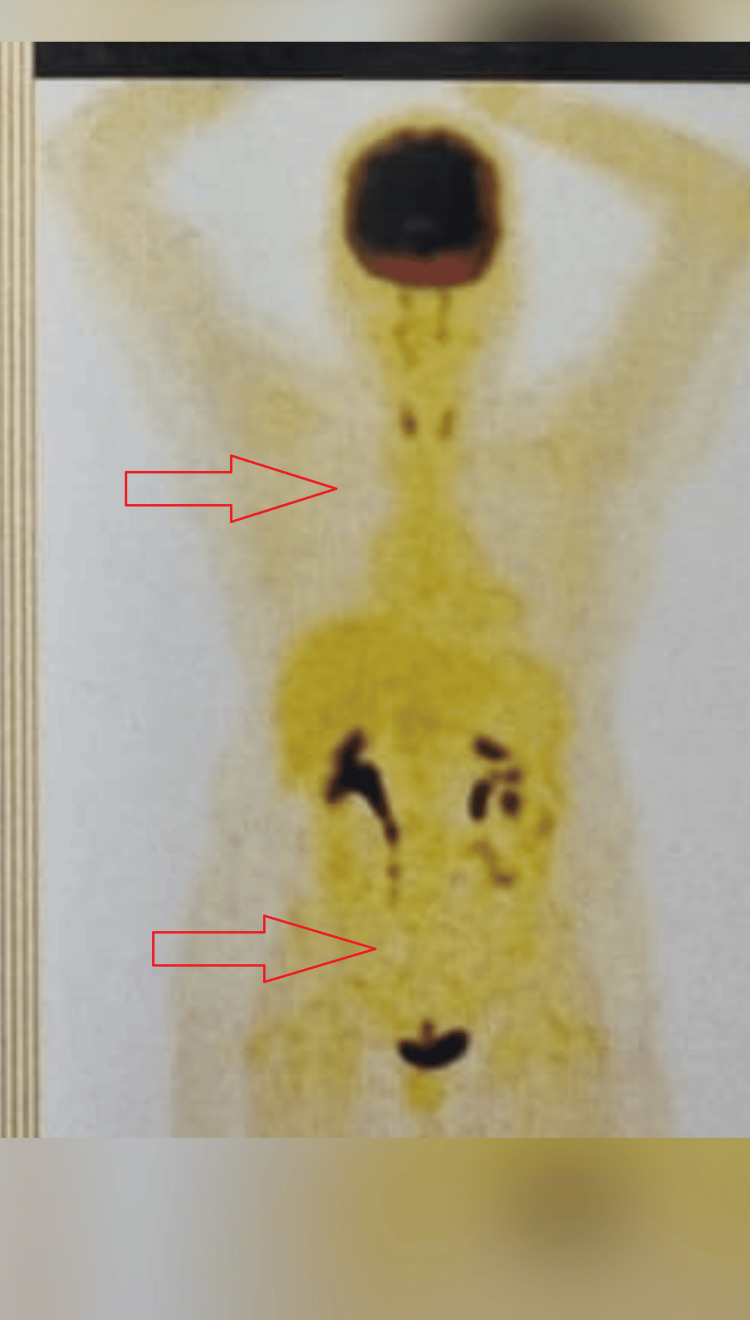
Annual follow-up positron emission tomography-computed tomography scan showing complete metabolic response.

Follow-up PET-CT done one year after chemotherapy showed no evidence of disease (Figure 4), and an echocardiogram showed an ejection fraction of 65%.

## Discussion

Cardiac masses arising from the heart are usually dangerous, whether defined as benign or malignant etiology. Approximately 75% are benign cardiac masses. Primary malignant tumors include sarcomas and lymphomas [2]. Secondary involvement includes metastatic deposits from the lung, esophagus, breast, lymphoma, leukemia, and melanoma [3]. Moreover, involvement by direct extension from mediastinal tumor, retrograde lymphatic spread, and hematogenous spread is also noted. Cardiac involvement is frequently undetected before death. Most of the cardiac involvement by lymphoma data have been retrieved from autopsy studies at 16% in Hodgkin’s lymphoma and 18% in non-Hodgkin’s lymphoma [4-6]. Clinical presentation of cardiac involvement is based on many factors, including tumor location, growth rate, size of the lesion, degree of invasion, and friability. The cardiac mass may obstruct blood flow, valve dysfunction, arrhythmias, pericardial effusion, cardiac tamponade, and tumor embolization. Our patient presented with dyspnea on exertion and chest discomfort. Imaging techniques increasingly identify cardiac involvement in lymphoma patients. Chest radiographs lack sensitivity and specificity but may identify cardiomegaly, heart failure features, and abnormality in heart contour. Echocardiogram can examine the heart chambers and pericardium with limitations due to the restricted acoustic window of the transthoracic approach [7]. Transesophageal echocardiogram remains a sensitive technique. CT images assess morphology, location, and tumor extension [8,9]. MRI can help identify anatomy, cardiac function, and blood flow [9]. Fluorodeoxyglucose PET is more accurate for evaluating lymphoma extension, including evaluating the cardiac masses and extracardiac tumor proliferation, and is helpful in staging and post-treatment follow-up [10,11]. Our patient had enlarged cervical, mediastinum, abdominal, and pelvic lymph nodes with residual cardiac involvement.

There have been many single case reports in the literature regarding the management of cardiac involvement patients due to the rarity and lack of definitive guidelines. The available literature shows systemic chemotherapy is the preferred option for R-CHOP /R-EPOCH chemotherapy. Because of low incidence and lack of clinical research studies, superior regimens to R-CHOP chemotherapy remain unclear. Although there is a theoretical risk for cardiac wall rupture, case reports in the literature have not reported any cardiac wall rupture. Resection of cardiac masses can be considered in hemodynamically unstable patients and right ventricle outflow obstruction. Our patient presented with a deterioration in her general condition and was taken up for emergency atrial mass resection. Post-resection, the patient received six cycles of R-CHOP chemotherapy. The response assessment PET-CT showed complete metabolic response (Deauville score 1). The patient is on regular follow-up every three months. Post-treatment annual follow-up PET-CT imaging showed complete response (Figure 3). Moreover, cardiac function assessment with echocardiogram showed normal ejection fraction and no cardiac dysfunction.

## Conclusions

Cardiac involvement by lymphoma is rarely described or easily missed in clinical practice. Because most lymphomas involving heart structure are secondary, the cardiac presentation is masked by other symptoms such as lymphadenopathy, spleen enlargement, fever, fatigue, weight loss, and drenching night sweats. Echocardiography should not be excluded in diagnostic examination. Cardiac lymphoma should be a part of the differential diagnosis of a space-occupying mass in the heart because it may lead to serious complications such as intracardiac obstruction and fatal arrhythmias. Multimodality imaging plays an important role in distinguishing cardiac masses to provide optimal treatment.
